# Knowledge Attitudes and Sense of Self-Efficacy of Primary Education Teachers towards Students with Insulin-Dependent Diabetes Mellitus

**DOI:** 10.1192/j.eurpsy.2023.2093

**Published:** 2023-07-19

**Authors:** D. Detsis, E. Vlachou, T. Adamakidou, A. Zartaloudi

**Affiliations:** 1 University of West Attica, Athens, Greece

## Abstract

**Introduction:**

The positive attitude towards students with Diabetes Mellitus type 1(DM1) and the teacher’s knowledge seem to be important conditions both for the practical support of children with type 1 ED in primary education and for the self-efficacy of teachers in the school context. Self-efficacy involves the belief that a person has the ability to create change through personal actions.

**Objectives:**

To investigate the level of knowledge of Primary Education teachers about DM1, their attitudes towards students with insulin-dependent diabetes, as well as their levels of self-efficacy in the management of diabetes in the school environment.

**Methods:**

This is a cross-sectional study, where the sample consisted of 150 teachers working in Public Primary Schools of Athens, the capital of Greece. The following questionnaires were used to collect the data: (a) socio-demographic characteristics questionnaire, (b) primary education teachers’ knowledge and attitudes questionnaire about insulin-dependent diabetes, and (c) the generalized self-efficacy scale.

**Results:**

24.7% of participants were male and the mean age was 43.79 (±10.11). The average percentage of correct answers to the knowledge questionnaire was 86.00 (±9.01), also, the average score of perceptions about the role of the school nurse was 90.53 (±9.39), for the role of students with DM1 89.17 (±11.42), for the readiness of teachers/educational system to manage students with DM1 48.10 (±13.22), and of the self-efficacy was 30.71 (±13, 67). The questions with the lowest percentage of correct answers were: “What is the normal range of blood sugar values when we are fasting?” (62.70%) and “If you find a child with DM1 unconscious what should you do immediately?” (52.70%). The percentage of correct responses was found to have a positive statistically significant correlation with self-efficacy score (p=0.05), years of service (p=0.003), age (p=0.014), teachers who had at some point students with DM1 in their classroom versus those who did not (p=0.045).

**Conclusions:**

Experienced and qualified staff could effectively support students not only practically by assisting them with daily activities but also by creating an environment based on understanding and acceptance.

**Disclosure of Interest:**

None Declared

